# Are the precapillary sphincters and metarterioles universal components of the microcirculation? An historical review

**DOI:** 10.1007/s12576-013-0274-7

**Published:** 2013-07-04

**Authors:** Tatsuo Sakai, Yasue Hosoyamada

**Affiliations:** 1 Department of Anatomy and Life Structure, Faculty of Medicine, Juntendo University, 2-1-1 Hongo, Bunkyo-ku, Tokyo, 113-8421 Japan; 2 Department of Nutrition, Faculty of Health Care Sciences, Chiba Prefectural University of Health Sciences, 2-10-1 Wakaba, Mihama-ku, Chiba-shi, Chiba, 261-0014 Japan

**Keywords:** Microcirculation, Mesentery, Precapillary sphincter, Metarteriole, Physiology textbook

## Abstract

The microcirculation is a major topic in current physiology textbooks and is frequently explained with schematics including the precapillary sphincters and metarterioles. We re-evaluated the validity and applicability of the concepts precapillary sphincters and metarterioles by reviewing the historical context in which they were developed in physiology textbooks. The studies by Zweifach up until the 1950s revealed the unique features of the mesenteric microcirculation, illustrated with impressive schematics of the microcirculation with metarterioles and precapillary sphincters. Fulton, Guyton and other authors introduced or mimicked these schematics in their physiology textbooks as representative of the microcirculation in general. However, morphological and physiological studies have revealed that the microcirculation in the other organs and tissues contains no metarterioles or precapillary sphincters. The metarterioles and precapillary sphincters were not universal components of the microcirculation in general, but unique features of the mesenteric microcirculation.

## Introduction

**Fig. 1:**
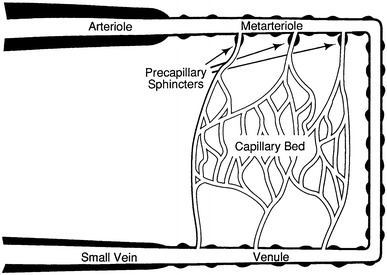
Schematic diagram of the microcirculation. From Johnson [1]

**Fig. 2:**
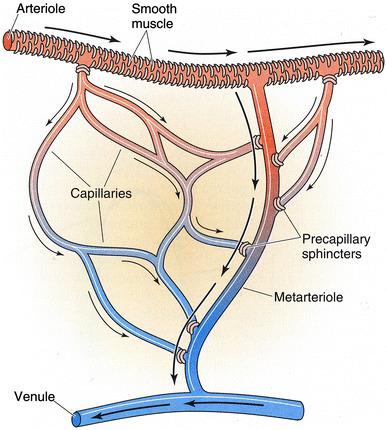
Idealized microcirculatory circuit. From Boron and Boulpaep [2]

The microcirculation is one of the major topics in the study of physiology and histology. In current physiology textbooks, the general architecture of the microcirculation is frequently explained with schematic drawings depicting precapillary sphincters and metarterioles in addition to arterioles, capillaries and venules. Judging from the main text and figures with legends in relatively recent textbooks such as Johnson’s [1] (Fig. 1) and Boron and Boulpaep’s [2] (Fig. 2), one could assume that the precapillary sphincters and metarterioles are universal components of the microcirculation in various tissues and organs of the human body.

Since the concept of precapillary sphincters and metarterioles is quite popular in physiology textbooks, one might expect that previous physiological investigations had revealed the existence of these structures in various tissues with reasonable certainty. Nevertheless, the existence of precapillary sphincters as universal components of the microcirculation has been questioned by some physiologists. McCuskey [3] pointed out the diversity of the structure and function of the microcirculation among various tissues and criticized the harsh generalization of precapillary sphincters. Wiedeman et al. [4] recommended the expression “precapillary resistance” instead of precapillary sphincters based on the absence of consensus on the definition of the precapillary sphincter and of supportive morphological findings. Furthermore, in the literature surveyed using PubMed, the metarterioles were frequently conceived in a restricted sense as representing the smallest and last order of arterioles instead of in their original sense of thoroughfare channels connecting arterioles and venules.

In the present article, we re-evaluated the validity and applicability of the concept of precapillary sphincters and metarterioles by reviewing the historical context in which these concepts were developed and accepted in four steps. In the first part, we investigated the historical process of how the schematics of these concepts was developed and incorporated into physiology textbooks. In the second part, we evaluated the validity of the concepts by examining the physiological literature on the microcirculation of the mesentery in which the concepts were originally proposed. In the third part, we examined the applicability of the concepts to the microcirculation of organs other than the mesentery. In the fourth part, we verified the structural counterparts of the concepts by examining the histological literature on the vasculature.

## The origin of the schematics of the microcirculation

We examined the available physiology textbooks from Haller’s “Primae Lineae Physiologiae” of 1747 [5] to the most modern “Medical Physiology” by Boron and Boulpaep [2] as well as histology textbooks from Kölliker’s “Handbuch der Gewebelehre des Menschen” of 1852 [6] to Ross and Pawlina’s “Histology” [7] to look for accounts and schematics of the microcirculation. The earliest example of a textbook containing an account and schematics of the microcirculation was “Howell’s Textbook of Physiology,” 15th edition, edited by Fulton [8], in which the schematics was reproduced from figure 1 of Zweifach’s 1937 article [9] on the mesentery of the frog without either precapillary sphincters or metarterioles. Fulton’s “A Textbook of Physiology,” 17th edition, published in 1955 [10], contained a new schematic of the ideal capillary bed containing both precapillary sphincters and metarterioles reproduced from figure 1 of Zweifach et al.'s 1953 publication [11]. Thereafter, the physiology textbooks usually contain an account of the microcirculation together with schematics showing both precapillary sphincters and metarterioles, as shown in Table 1. Recent histology textbooks, such as Ross and Reith’s histology published in 1985 [12] and its later versions, contain an account and schematics of the microcirculation.

**Table 1:** Features of the schematics of the microcirculation in the physiology textbooks

*Fulton “Textbook of Physiology”*		
Fulton: Howell’s Textbook of Physiology. 15th ed., 1946	MA (−)	PS (−)
Fulton: Textbook of Physiology. 16th ed., 1949	MA (−)	PS (−)
Source: Zweifach [9], figure 1 (Fig. 3 in the present article)		
Legends: capillaries, a–v capillaries and their relations to arterioles and venules		
Credit: from Zweifach, Amer. J. Anat., 1936–1937, 60: 473–714		
Fulton: Textbook of Physiology. 17th ed., 1955	MA (+)	PS (+)
Source: Zweifach et al. [11], figure 1 (Fig. 8 in the present article)		
Legends: schematic diagram of ideal capillary bed		
Credit: according to Chambers and Zweifach		
*Guyton “Textbook of Medical Physiology”*		
Guyton: Textbook of Medical Physiology. 1st ed., 1956	MA (+)	PS (+)
Source: Chambers and Zweifach [17], figure 1 (Fig. 6 in the present article)		
Legends: functional anatomy of the capillaries		
Credit: redrawn from Chambers, R., and Zweifach, B.W.: Am. J. Anat, 75: 179, 1944		
Guyton: Textbook of Medical Physiology. 2nd–4th ed., 1961–1971	MA (+)	PS (+)
Source: Zweifach [13], figure 2 (Fig. 7 in the present article)		
Legends: overall structure of capillary bed		
Credit: from Zweifach: Factors regulating blood pressure, Josiah Macy, Jr. Foundation, 1950		
Guyton: Textbook of Medical Physiology. 6th–9th ed., 1981–1996	MA (+)	PS (+)
Source: Zweifach [13], figure 2 (Fig. 7 in the present article)		
Legends: structure of mesenteric capillary bed		
Credit: from Zweifach: Factors regulating blood pressure, Josiah Macy, Jr. Foundation, 1950		
Guyton: Textbook of Medical Physiology. 10th–11th ed., 2000–2005	MA (+)	PS (+)
Source: Zweifach [13], figure 2 (Fig. 7 in the present article)		
Legends: structure of mesenteric capillary bed		
Credit: redrawn from Zweifach: Factors regulating blood pressure, Josiah Macy, Jr. Foundation, 1950		
*Berne and Levy “Cardiovascular Physiology”*		
Berne and Levy: Cardiovascular Physiology. 1st–4th ed., 1967–1981	MA (+)	PS (+)
Source: original drawing (Fig. 9 in the present article)		
Legends: schematic drawing of the microcirculation		
Credit: after Zweifach		
Berne and Levy: Cardiovascular physiology. 5th ed., 1986	MA (+)	PS (+)
Source: original drawing (Fig. 9 in the present article)		
Legends: schematic drawing of the microcirculation		
Credit: none		
Berne and Levy: Cardiovascular Physiology. 6th–9th ed., 1992–2002	MA (+)	PS (−)
Source: original drawing (Fig. 9 in the present article)		
Legends: composite schematic drawing of the microcirculation		
Credit: none		
*Bard/Mountcastle “Medical Physiology”*		
Bard: Medical Physiology. 11th ed., 1961		
Mountcastle: Medical Physiology. 14th ed., 1980		
Fig. 5 (1961), Fig. 43-1 (1980)	MA (+)	PS (+)
Source: Zweifach [13], figure 1 (Fig. 5 in the present article)		
Legends: camera lucida drawing of the microcirculation in mesentery of cat		
Credit: From Zweifach: Third conference on factors regulating blood pressure, New York, 1950, Josiah Macy, Jr. Foundation		
Fig. 6 (1961), Fig. 43-2 (1980)	MA (−)	PS (−)
Source: Zweifach [13], figure 4 (Fig. 4 in the present article)		
Legends: camera lucida drawing of capillary bed in mesentery of dog		
Credit: From Zweifach: Third conference on factors regulating blood pressure, New York, 1950, Josiah Macy, Jr. Foundation		
*Johnson “Essential Medical Physiology”*		
Johnson: Essential Medical Physiology. 1st–3rd ed., 1992–2003	MA (+)	PS (+)
Source: original drawing (Fig. 1 in the present article)		
Legends: schematic diagram of the microcirculation		
Credit: none		
*Boron and Boulpaep “Medical Physiology”*		
Boron and Boulpaep: Medical Physiology. 1st–2nd ed., 2003–2008	MA (+)	PS (+)
Source: original drawing (Fig. 2 in the present article)		
Legends: idealized microcirculation circuit		
Credit: none		
*Ross et al. “Histology”*		
Ross and Reith: Histology. 1st ed., 1985	MA (+)	PS (+)
Ross, Kaye and Pawlina: Histology. 4th ed., 2003	MA (+)	PS (+)
Source: Zweifach et al. [11], figure 1 (Fig. 8 in the present article)		
Legends: diagram of the microcirculation		
Credit: courtesy of Zweifach		
Ross and Pawlina: Histology. 5th ed., 2006	MA (+)	PS (+)
Source: Zweifach et al. [11], figure 1 with modification (Fig. 8 in the present article)		
Legends: diagram of the microcirculation		
Credit: none		

*MA* Meta-arterioles, *PS* precapillary sphincters

The schematics of the microcirculation in the textbooks shown in Table 1 were either reproduced from a few of Zweifach’s articles or drawn without any indication of the source. We found that six different schematics from four of Zweifach’s articles were used as the source in the textbooks.

**Fig. 3:**
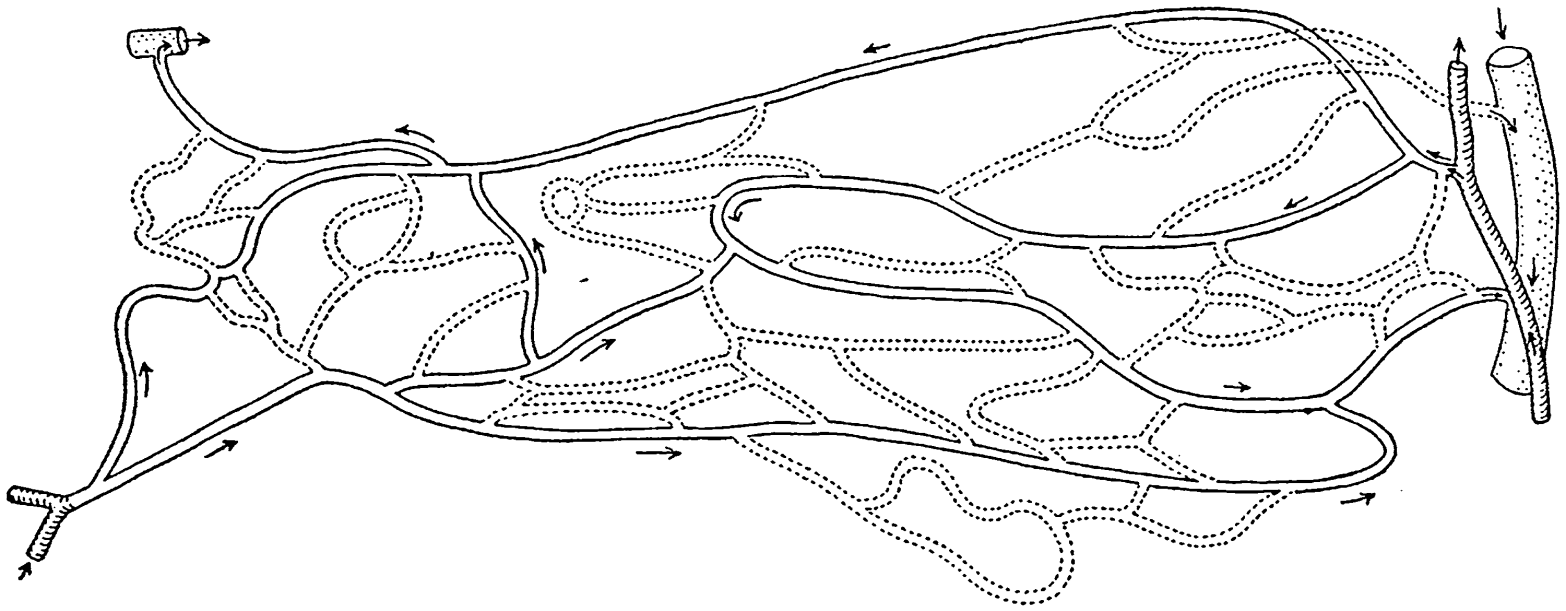
Camera lucida outline of vessels in the capillary bed of the frog mesentery. Figure 1 from Zweifach [9]

**Fig. 4:**
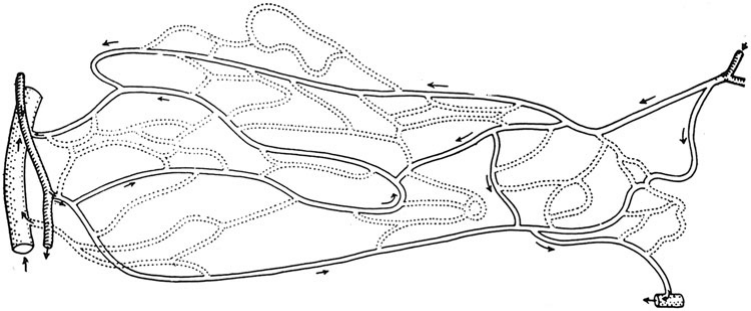
Camera lucida drawing of the capillary bed in mesentery of dog. Figure 4 from Zweifach [13]

Among the six schematics, two figures contained neither precapillary sphincters nor metarterioles, including figure 1 from Zweifach’s 1937 article [9] on the frog mesentery (Fig. 3) and figure 4 from Zweifach’s 1950 article [13] on the dog mesentery (Fig. 4). Strangely, both figures were identical except that the latter was rotated upside-down. The former was reproduced in Fulton’s physiology in 1946 [8] and 1949 [14], and the latter was reproduced in Bard’s physiology in 1961 [15] and its later versions until Mountcastle’s physiology in 1980 [16].

**Fig. 5:**
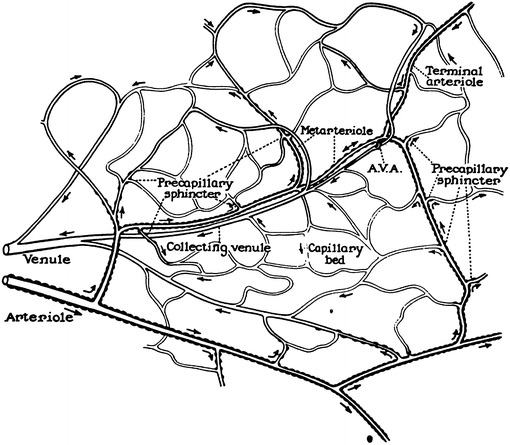
Camera lucida drawing of the capillary bed in the mesentery of the cat. Figure 1 from Zweifach [13]

One of the six figures showed sketches of observations of the mesentery microcirculation with precapillary sphincters and metarterioles, namely figure 1 in Zweifach’s 1950 article [13] (Fig. 5). This figure was reproduced in Bard’s physiology in 1961 [15] and its later versions up until Mountcastle’s physiology in 1980 [16].

**Fig. 6:**
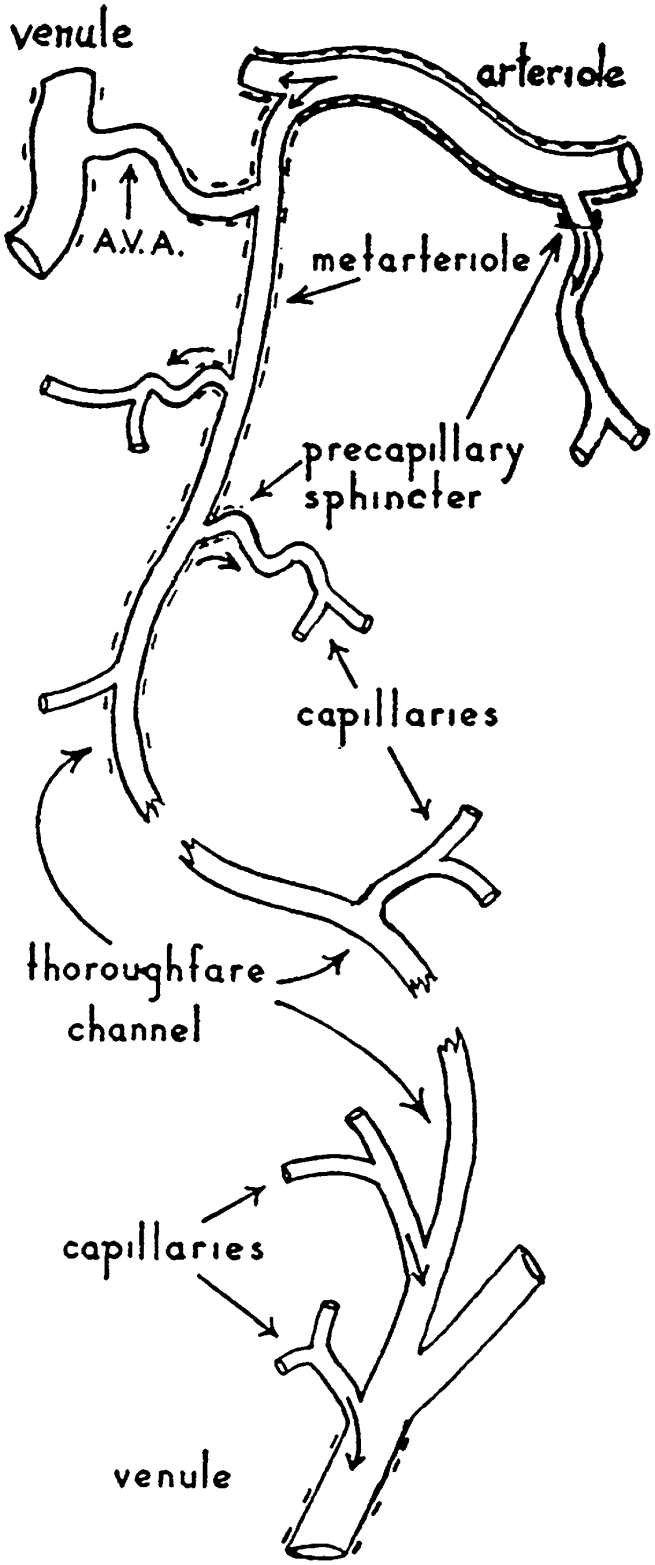
Diagram of a functional unit of the capillary bed. Figure 1 from Chambers and Zweifach [17]

**Fig. 7:**
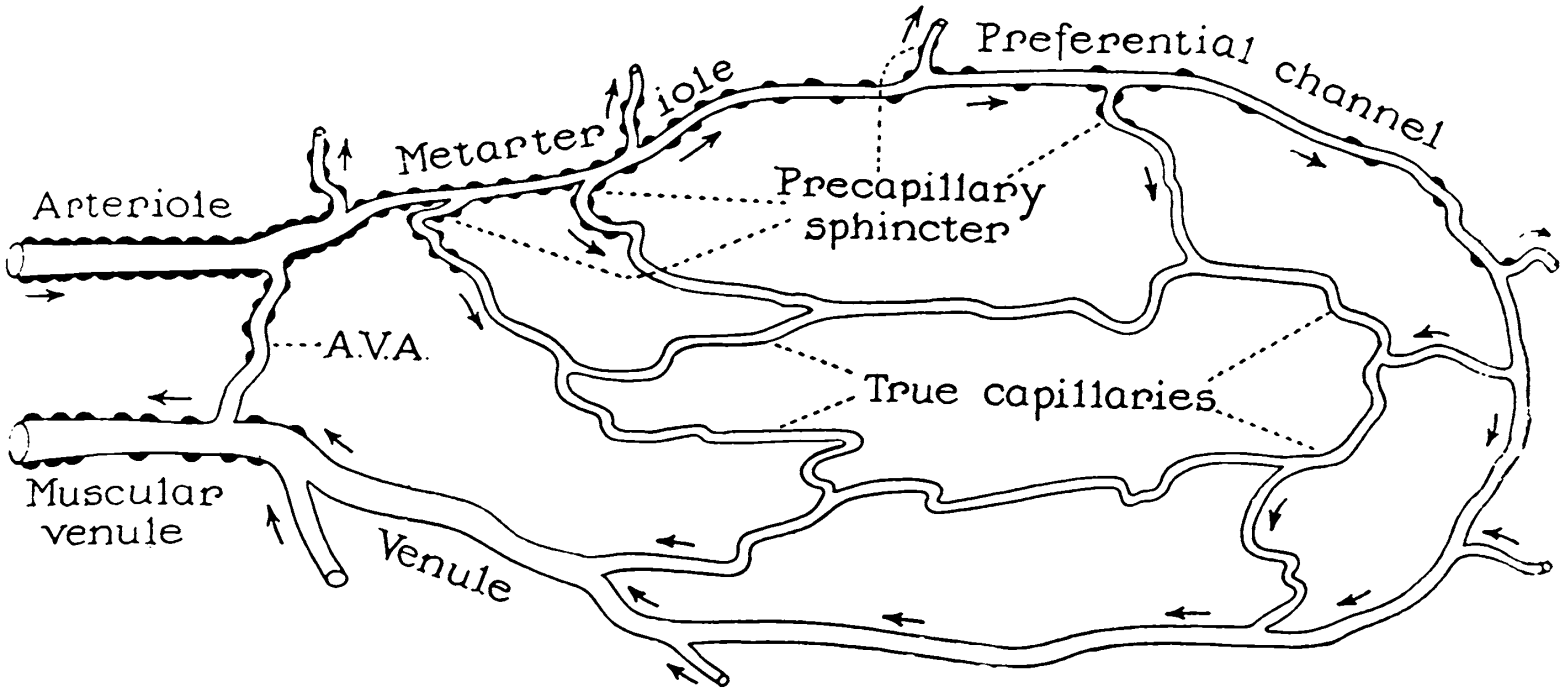
A schematic representation of the structural pattern of the capillary bed. Figure 2 from Zweifach [13]

**Fig. 8:**
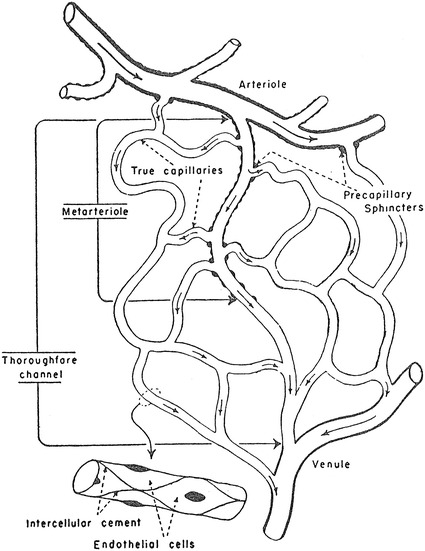
Diagrammatic representation of basic structural pattern of the terminal vascular bed as visualized in the mesentery. Figure 1 from Zweifach et al. [11]

The other three figures represented an idealized microcirculation and contained both precapillary sphincters and metarterioles, including figure 1 of Chambers and Zweifach’s article from 1944 [17] (Fig. 6), figure 2 of Zweifach’s article from 1950 [13] (Fig. 7) and figure 1 of Zweifach et al. from 1953 [11] (Fig. 8). The first one was reproduced in Guyton’s 1956 physiology book [18], the second in Guyton’s 1961 physiology book [19] and its later versions, and the third in Fulton’s 1955 physiology book [10] and Ross and Reith’s 1985 histology [12] and its later versions.

**Fig. 9:**
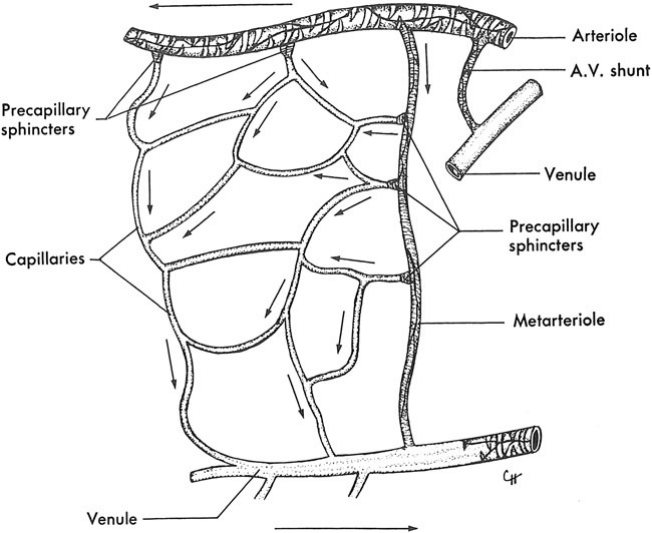
Schematic drawing of the microcirculation (after Zweifach). From Berne and Lavy [20]

The other schematics of the microcirculation in the physiology textbooks did not use Zweifach’s article as the source for the figures and appeared to be original drawnings. The schematic in Berne and Levy’s physiology in 1967 [20] and its later versions (Fig. 9) showing both precapillary sphincters and metarterioles was credited as being taken from Zweifach of unknown origin, but it may have been an original drawing since no similar figures are found in Zweifach’s literature, and the same schematic was used in Berne and Levy’s physiology textbook in 1986 [21] and its later versions without any reference to the source. The schematics in Johnson’s 1992 book on physiology [22] and its later versions (Fig. 1) and Boron and Boulpaep’s book on physiology in 2003 [23] and the second edition (Fig. 2) with both precapillary sphincters and metarterioles were newly drawn without any particular source indicated.

The above survey indicated that the schematics of the microcirculation in the current textbooks of physiology relied on specific articles by Zweifach before 1953. In the following part, we will examine the literature on physiological research on the microcirculation, especially those by Zweifach, to evaluate the extent of applicability of the concepts of precapillary sphincters and metarterioles.

## Physiological research as the basis of precapillary sphincters and metarterioles

A series of investigations by Zweifach utilized the mesentery of a few different animals for visualization of the microcirculation in vivo, beginning with Zweifach’s research in 1937 [9] on the frog mesentery. In this article Zweifach observed the microcirculation in both living and fixed material and found neither precapillary sphincters nor metarterioles, as shown in the camera lucida outline of the capillary bed (Fig. 3). Two years later, in 1939, Zweifach [24] reported the first indication of precapillary sphincters in the rabbit and mouse mesentery by describing a valve-like fold comprised of endothelium at the point where a capillary branch leaves an arteriole. He stated that the folds behaved like an endothelial sphincter when the parent trunk contracted, but further stated that in arterioles, closure was aided by muscle cells at the point of capillary exit. In 1942 Chambers and Zweifach [25] presented a motion picture that showed “sphincter like functioning of the precapillaries at their junctions with the arteriole.” In 1944, Chambers and Zweifach [17] presented a diagram of a functional unit of the capillary bed in which thoroughfare vessels with preferentially rapid blood flow were designated as metarterioles and a specific area at the origin of capillaries as precapillary sphincters, as shown in a diagram of the functional unit of capillary bed (Fig. 6).

In introducing the new concepts of metarterioles and precapillary sphincters, Chambers and Zweifach [17] referred to previous investigations to support their conclusions. They claimed that the two different vascular components observed by Sandison [26] and by Clark and Clark [27] in the rabbit ear either with or without contractile activity corresponded to the metarterioles and capillaries in the mesentery. However, the vascular components with contractile activity in the rabbit ear were different from metarterioles, since the metarterioles as proposed by Chambers and Zweifach [17] represented thoroughfare channels connecting arterioles and venules. Chambers and Zweifach [17] claimed that the contractile mechanism observed at the arteriolar source of the capillaries in three previous studies, including Richards and Schmidt’s [28] research on the glomerular capillaries of the frog, Crawford’s [29] research on the epidermal papillae at the base of the human finger, and Fulton and Lutz’s [30] study on frog retrolingual membrane, was so variable and ambiguous that the specific vasoconstriction at the origin of the capillaries could not be determined and that the last one described the precapillary contractility.

Summarizing the above literature survey, it must be concluded that the precapillary sphincters and metarterioles identified by several studies by Zweifachs provide enough evidence on the mesentery microcirculation, but their findings cannot be accepted as evidence for the microcirculation of the various tissues and organs in general.

Zweifach provided four types of schematics including precapillary sphincters and metarterioles in three articles. The first type was in an article on the mammalian omentum and mesentery by Chambers and Zweifach in 1944 [17] presenting a diagram of the functional unit of the capillary bed (Fig. 6), while the second and third types were published in a conference report by Zweifach in 1950 [13] either as a camera lucida drawing of the capillary bed in the mesentery of a cat or as a schematic representation of the structural pattern of the capillary bed (Figs. 5, 7). The fourth type was described by Zweifach et al. in 1953 [11] in an article on the influence of the adrenal cortex on the microcirculation as a diagrammatic representation of the basic structural pattern of the terminal vascular bed as visualized in the mesentery (Fig. 8). Zweifach clearly recognized that evidence on the precapillary sphincters and metarterioles was only available in the mesentery, but obviously wanted to present them as universal elements of the microcirculation.

However, the metarterioles and precapillary sphincters were topics of later physiological research on the microcirculation of various tissues and organs including the skeletal muscles [31–39], skin [40, 41], subcutaneous adipose tissue [42], gastric mucosa [43], liver [44] and heart [45]. In these studies the metarterioles and precapillary sphincters obviously differed from the original concepts by Zweifach. In these studies, the metarterioles were conceived in a restricted sense as representing the smallest and last order of arterioles [41, 45], and they were fundamentally different from the thoroughfare channels connecting arterioles and venules as originally proposed by Zweifach [11, 13, 17] and accepted in modern physiology textbooks [1, 2, 10, 15, 16, 18–23]. Furthermore, in these studies the precapillary sphincters were not substantially demonstrated at specific portions of the microcirculation, but the change in vascular resistance was interpreted as effected by “precapillary sphincter tone” or “precapillary sphincter activity” [34–39, 43], or the last segments of the arterial tree before capillaries were merely designated as the precapillary sphincters without showing any specific localization of smooth muscle cells [31–33, 40, 42, 44]. As Wiedeman et al. [4] recommended, the expression of “precapillary resistance” instead of precapillary sphincters may be preferable when structural evidence is not available. It would be appropriate to conclude that the concepts of precapillary sphincters and metarterioles are diverse mainly because of the difference in definitions adopted by recent physiological researchers from those originally proposed by Zweifach and accepted in the current physiology textbooks. In addition, the difference in definitions was blurred by the structural and functional heterogeneity of the microcirculation among tissues and organs, as will be discussed in the next section.

## The architecture of the microcirculation is diverse and specific to organs

After the pioneering studies of Zweifach and coworkers, the structure and function of the microcirculation were studied in other organs such as the skeletal muscle, skin, heart, liver, kidney, intestine, etc. The microcirculation architecture was revealed to be quite diverse among organs and had specific patterns suitable to the functional demands of the individual organs.

In skeletal muscle, the arterioles and venules branch successively to become the terminal segments that supply and drain microvascular units, respectively [46]. The terminal segments of arterioles give rise to a group of capillaries to form a microvascular unit, with respective capillaries converging on collecting venules. Between the arterioles and venules, there are no preferential channels representing the metarterioles. The total flow into a muscle is governed by the whole arterial tree from the feeding arteries to the terminal arteries. The specific precapillary sphincters at the junction of arterioles are not present in the microcirculation of skeletal muscles. The microcirculation in the skeletal muscle is arranged to distribute the blood flow evenly to the individual muscle fibers and to modulate the blood flow to meet the increased demand during exercise.

In the skin, the microcirculation is organized as two horizontal plexuses [47]. One is situated 1–1.5 mm below the skin surface, and the other is at the dermal-subcutaneous junction. Ascending arterioles and descending venules are paired as they connect the two plexuses. The lower plexus is formed by perforating vessels from the underlying muscles and subcutaneous fat, and it supplies the hair bulbs and sweat glands. The superficial horizontal plexus supplies capillaries that course close to the dermal-epidermal junction and also serve as a thermal radiator.

In the coronary microcirculation, the small penetrating arteries give rise to arterioles at almost right angles, which take either longitudinal or oblique courses to the muscle fibers to give off capillaries around the muscle fibers [48]. Between the arterioles and venules, there are no preferential channels representing the metarterioles. The coronary resistance is considered to be primarily regulated by arterioles with diameters of 50–200 μm, which are most responsible for metabolic, myogenic and humoral stimuli [49]. The coronary microcirculation is arranged to distribute the blood flow evenly to the cardiac muscle cells and to modulate the blood flow to meet the increased demand for high cardiac output.

In the liver, the hepatic arteries (HAs) and portal veins (PVs) divide successively into terminal HAs and PVs in the connective tissue stroma at the periphery of the hepatic lobules. The terminal PVs directly supply the sinusoids, while the terminal HAs drains into the terminal PVs and the proximal part of the sinusoids. The sinusoidal blood is drained via central venules into the hepatic veins [50]. There are neither metarterioles nor precapillary sphincters in the liver. The hepatic microcirculation is thought to be suitable for handling nutrients absorbed in the intestine with the hepatocytes around the sinusoids.

In the kidney, the arteries enter the parenchyme and divide into the arcuate arteries at the corticomedullary boundary from which the interlobular arteries arise toward the cortical surface to branch off successively into the afferent arterioles to supply the glomerular capillaries in the individual glomeruli [51]. The efferent arterioles drain the individual glomeruli to pour into the peritubular capillaries, which drain into branches of the renal veins. There are neither metarterioles nor precapillary sphincters in the kidney. The renal microcirculation is thought to be suitable for large amounts of glomerular filtration together with reabsorption of most of the fluids in the renal tubule.

In the small intestine, the arterial and venous plexuses are formed in the submucosa [52]. From the submucosal arterial plexus, a single arteriole arises to supply the intestinal villus to reach the tip of the villus and breaks up into a fountain-like pattern of capillaries. These capillaries drain into the villus venules, which pass straight down to enter the submucosal venous plexus. There are neither metarterioles nor precapillary sphincters in the intestinal wall. The intestinal microcirculation is thought to be suitable for reabsorption of fluids and nutrients in the intestinal villi.

However, in the mesenteric microcirculation, the metarterioles have been recognized by the other authors as shunting arterioles [53]. The blood flow through the arteriovenous shunt is reported to be 2 or 3 % of the total mesenteric blood flow [54, 55].

The metarterioles in the mesentery represent thoroughfare channels between the arterioles and venules and therefore can be regarded as a kind of arteriovenous anastomosis. These have been reported in various tissues and organs including the skin, skeleton, muscle, lung, heart, intestinal canal, kidney, brain, eye and ear [56]. However, a systematic survey in various organs reported that substantial arteriovenous anastomoses are present only in the ears and skin and absent in the brain, heart, kidneys, skeletal muscles, stomach, small and large intestines, spleen, adrenal glands, liver, bones, fat and salivary glands [57]. In the skin, arteriovenous anastomoses are known to have specific physiological functions to prevent lowering of the body temperature [47, 58]. In pathological conditions, arteriovenous anastomoses in the gastric mucosa can cause local ischemia and prevent bleeding from a gastric ulcer [59]. The metarterioles in the mesentery are structurally and functionally clearly distinguished from the representative arteriovenous anastomoses in the skin.

## Morphological observations on the microvasculature

The structure of the microvasculature has been repeatedly investigated in various tissues by means of three electron microscopy methods. By transmission electron microscopy (TEM), the smooth muscle cells and extracellular matrices of the vascular wall can be well visualized and analyzed, but it is usually difficult to identify the observed location within the three-dimensional branching of the vascular tree on the sectional planes. Thus, identification of precapillary sphincters may only be possible after repeated careful observations of the microvasculature with TEM. By scanning electron microscopy of vascular casts (SEM-vc), the three-dimensional branching pattern of the vasculature can be well visualized and analyzed, but it was practically impossible to identify the cellular composition of the vascular wall. The precapillary sphincter can be inferred by constriction of the vascular casts at the base of the vascular branches with SEM-vc. By scanning electron microscopy after removal of the extracellular matrices (SEM-rem), both the structure of the vascular wall and the three-dimensional branching pattern can be well visualized, but the cellular types can be identified only tentatively on the basis of cellular shape. The precapillary sphincters can be identified convincingly with SEM-rem.

In a search of the literature, we found 36 morphological studies on the microvasculature in 16 kinds of tissues employing one of the three morphological methods (Table 2). Six of the studies described precapillary sphincters, two employing TEM [60, 61] and four employing SEM-vc [62–66]. In the other studies, the existence of precapillary sphincters was neither mentioned in the text nor demonstrated in the figures; in some studies their absence was concluded. To evaluate the existence of precapillary sphincters, we scrutinized the descriptions and figures of the six studies reporting precapillary sphincters.

**Table 2:** Morphological studies on the microvasculature in various tissues and organs with three different methods

	TEM	SEM-vc	SEM-rem
Skin	Higgins and Eady [86]		
Skeletal muscle	Stingl et al. [87]		Holley and Fahim [88]
Muscle fascia	Rhodin [60]		
Brain		Nakai et al. [64] Castenholz [65]	Shiraishi et al. [69] Ushiwata and Ushiki [70]
Retina		Risco et al. [89] Morrison et al. [90] Yoneya et al. [91] Pannarale et al. [92, 93] Bhutto and Amemiya [94–96]	Murakami et al. [97]
Strial vessel/inner ear			Nagai et al. [98]
Dental pulp			Zhang et al. [99] Iijima and Zhang [100]
Heart	Sherf et al. [61] He et al. [66]	Anderson and Anderson [63] Tsunenari [67]	Higuchi et al. [68]
Lung	Lane et al. [101] Sasaki et al. [71]		
Mammary gland			Fujiwara and Uehara [102]
Small intestine	Hosoyamada et al. [75, 76]	Anderson and Anderson [63]	Miller et al. [103]
Gallbladder		Ohtani et al. [104]	Ohtani et al. [104]
Kidney	Sakai and Kriz [72] Elger et al. [73] Hosoyamada and Sakai [74]		
Epididymis		Pais and Esperança-Pina [105]	
Seminal cord		Polguj et al. [106]	
Retrolingual membrane/frog	Berman et al. [107]		

*TEM* Transmission electron microscopy, *SEM-vc* scanning electron microscopy of vascular casts, *SEM-rem* scanning electron microscopy after removal of extracellular matrices

Rhodin made detailed observations of the microvasculature employing TEM [60]. The identification of the observation site was accurate enough, since he selected the observation site in the whole-mount specimens of the muscular fascia. He identified the precapillary sphincter at the angle of small vessel branching, but the figures showed smooth muscle cells present not only at the angle but also in the walls of both the trunk vessels and the branches. The precapillary sphincters reported by Rhodin [60] were not separate smooth muscle cells at the angle of vascular branching, but a part of the continuous smooth muscle layer at the angle, so that they did not deserve the name precapillary sphincters.

Precapillary sphincters were reported in the microvasculature of the heart with TEM and SEM-vc. Sherf et al. [61] described precapillary sphincters with TEM in the human heart, but the electron micrographs did not allow determination of the location of smooth muscle cells in the three-dimensional vascular tree, so that the identification of precapillary sphincters was groundless and doubtful. Anderson and Anderson reported an indication of precapillary sphincters in dog heart with SEM-vc [63], but the abrupt interruption and irregular shape of the vascular casts indicated that the resin did not sufficiently fill the vascular lumina so that the identification of precapillary sphincters was not based on sound evidence. He et al. [66] reported step-wise constriction of arterioles in yaks and interpreted this as the precapillary sphincters being distributed in a certain section of the arterioles. These structures were far from the precapillary sphincters branching into capillaries reported in the physiological studies. The precapillary sphincters were not mentioned in the other studies on the microvasculature of the heart, including Tsunenari’s [67] research with TEM and Higuchi et al.’s [68] study with SEM-rem.

Precapillary sphincters were reported in the microvasculature of the brain with SEM-vc by Nakai et al. [64] and Castenholz [65]. Observations with SEM-vc did not provide conclusive evidence of precapillary sphincters, as mentioned above. However, Shiraishi et al. [69] did not mention the precapillary sphincters, and Ushiwata and Ushiki [70] denied the existence of precapillary sphincters in the brain microvasculature with the more reliable method of SEM-rem.

As shown above, the morphological evidence did not verify the existence of precapillary sphincters in the microvasculature of the muscular fascia, heart and brain. In the other 13 kinds of tissues, the precapillary sphincters were not observed at all with TEM, SEM-vc or SEM-rem. Our own observations of the microvasculature in the lung [71], kidney [72–74] and intestine [75, 76] did not show any indication of precapillary sphincters.

The arterioles received an abundance of aminergic (adrenergic) innervation in many organs such as the skeletal muscles [77–80], salivary glands [81], nasal mucosa [82], gastric and intestinal mucosa [83, 84], and brain [85]. Histochemical studies of adrenergic nerves in these organs can provide a good indication of smooth muscles in the walls of arteries and arterioles, but did not show the existence of precapillary sphincters [77, 78, 80–85]. On the whole, we should conclude that no morphological evidence has been obtained to support the existence of precapillary sphincters in all the tissues so far investigated.

## Conclusion

The studies by Zweifach up until the 1950s revealed the unique features of the mesenteric microcirculation and provided impressive schematics of the microcirculation with metarterioles and precapillary sphincters. Fulton, Guyton and other authors introduced or mimicked these schematics in their physiology textbooks as representative of the microcirculation in general. However, morphological studies have revealed that the microcirculation in other organs and tissues contains no metarterioles or precapillary sphincters, and physiological studies on the microcirculation have used the terms metarterioles and precapillary sphincters differently. This reveals that the metarterioles and precapillary sphincters are not universal components of the microcirculation in general, but unique features of the mesenteric microcirculation. Therefore, explanations and illustrations of the microcirculation with metarterioles and precapillary sphincters can be regarded as inappropriate and misleading in physiology textbooks about the organs and tissues that aim to teach in general and not specific terms.

## References

[1] Johnson LR (2003). Essential medical physiology.

[2] Boron WF, Boulpaep EL (2008). Medical physiology.

[3] McCuskey RS (1971). Sphincters in the microvascular system. Microvasc Res.

[4] Wiedeman MP, Tuma RF, Mayrovitz HN (1976). Defining the precapillary sphincter. Microvasc Res.

[5] Haller AV (1747). Primae lineae physiologiae in usum praelectionum academicarum.

[6] Kölliker R (1852). Handbuch der Gewebelehre des Menschen für Aerzte und Studierende.

[7] Ross MH, Pawlina W (2010). Histology. A text and atlas with correlated cell and molecular biology.

[8] Fulton JF (1946). Howell’s textbook of physiology.

[9] Zweifach BW (1937). The structure and reactions of the small blood vessels in Amphibia. Am J Anat.

[10] Fulton JF (1955). A textbook of physiology.

[11] Zweifach BW, Shorr E, Black MM (1953). The influence of the adrenal cortex on behavior of terminal vascular bed. Ann N Y Acad Sci.

[12] Ross MH, Reith EJ (1985). Histology. A text and atlas.

[13] Zweifach BW (1950) Basic mechanisms in peripheral vascular homeostasis. In: Zweifach BW (ed) Factors regulating blood pressure. Transactions of the third conference May 5–6, 1949, Josiah Macy, Jr. Foundation, New York, NY, pp 13–52

[14] Fulton JF (1949). A textbook of physiology.

[15] Bard P (1961). Medical physiology.

[16] Mountcastle VB (1980). Medical physiology.

[17] Chambers R, Zweifach BW (1944). Topography and function of the mesenteric capillary circulation. Am J Anat.

[18] Guyton AC (1956). Textbook of medical physiology.

[19] Guyton AC (1961). Textbook of medical physiology.

[20] Berne RM, Levy MN (1967). Cardiovascular physiology.

[21] Berne RM, Levy MN (1986). Cardiovascular physiology.

[22] Johnson LR (1992). Essential medical physiology.

[23] Boron WF, Boulpaep EL (2003). Medical physiology.

[24] Zweifach BW (1939). The character and distribution of the blood capillaries. Anat Rec.

[25] Chambers R, Zweifach BW (1942). Caliber changes of the capillary bed. Fed Proc.

[26] Sandison JC (1932). Contractions of blood vessels and observations on the circulation in the transparent chamber of the rabbit’s ear. Anat Rec.

[27] Clark ER, Clark EC (1943). Caliber changes in minute blood vessels observed in the living mammal. Am J Anat.

[28] Richards AN, Schmidt CF (1924). A description of the glomerular circulation in the frog’s kidney and observations concerning the action of adrenalin and various other substances upon it. Am J Physiol.

[29] Crawford JH (1926). Studies on human capillaries. II. Observations on the capillary circulation in normal subjects. J Clin Invest.

[30] Fulton GP, Lutz BR (1940). The neuro-motor mechanism of the small blood vessels of the frog. Science.

[31] Mellander S (1966). Comparative effects of acetylcholine, butyl-nor-synephrine (Vasculat), noradrenaline, and ethyl-adrainol (Effonti) on resistance, capacitance, and precapillary sphincter vessels and capillary filtration in cat skeletal muscle. Angiologica.

[32] Järhult J (1971). Comparative effects of angiotensin and noradrenaline on resistance, capacitance, and precapillary sphincter vessels in cat skeletal muscle. Acta Physiol Scand.

[33] Owen DA, Stuermer E (1971). Effect of dihydroergotamine (DHG) on the capacitance, resistance and precapillary sphincter vessels of denervated cat skeletal muscle. Br J Pharmacol.

[34] Lundvall J, Järhult J (1976). Beta adrenergic dilator component of the sympathetic vascular response in skeletal muscle. Influence on the micro-circulation and on transcapillary exchange. Acta Physiol Scand.

[35] Lundvall J, Hillman J (1978). Fluid transfer from skeletal muscle to blood during hemorrhage. Importance of beta adrenergic vascular mechanisms. Acta Physiol Scand.

[36] Hillman J, Lundvall J (1981). Classification of beta-adrenoceptors in the microcirculation of skeletal muscle. Acta Physiol Scand.

[37] Sacks FM, Dzau VJ (1986). Adrenergic effects on plasma lipoprotein metabolism. Speculation on mechanisms of action. Am J Med.

[38] Gustafsson D (1987). Microvascular mechanisms involved in calcium antagonist edema formation. J Cardiovasc Pharmacol.

[39] Bentzer P, Kongstad L, Grande PO (2001). Capillary filtration coefficient is independent of number of perfused capillaries in cat skeletal muscle. Am J Physiol.

[40] Tooke JE (1980). A capillary pressure disturbance in young diabetics. Diabetes.

[41] Widmer RJ, Laurinec JE, Young MF, Mohiddin MW, Laine GA, Quick CM (2008). The origin of the biphasic flow response to local heat in skin. Microcirculation.

[42] Burcher E, Olgart L, Gazelius B (1977). Comparative effects of adrenaline and felypressin (octapressin) on consecutive sections of the vascular bed in canine adipose tissue. Acta Physiol Scand.

[43] Perry MA, Granger DN (1985). Regulation of capillary exchange capacity in the dog stomach. Am J Physiol.

[44] Oda M, Han JY, Yokomori H (2000). Local regulators of hepatic sinusoidal microcirculation: recent advances. Clin Hemorheol Microcirc.

[45] Schneeweiss A, Sherf L, Lehrer E, Lieberman Y, Neufeld HN (1982). Segmental study of the terminal coronary vessels in coarctation of the aorta: a natural model for study of the effect of coronary hypertension on human coronary circulation. Am J Cardiol.

[46] Segal SS (2005). Regulation of blood flow in the microcirculation. Microcirculation.

[47] Braverman IM (1997). The cutaneous microcirculation: ultrastructure and microanatomical organization. Microcirculation.

[48] Kassab GS, Rider CA, Tang NJ, Fung YC (1993). Morphometry of pig coronary arterial trees. Am J Physiol.

[49] Beyer AM, Gutterman DD (2012). Regulation of the human coronary microcirculation. J Mol Cell Cardiol.

[50] Wanless IR, Shiff ER, Sorrell MF, Maddrey WC (2007). Physioanatomic considerations. Schiff’s diseases of the liver, vol 1.

[51] Kriz W, Kassling B, Seldin DW, Giebisch G (2000). Structural organization of the mammalian kidney. The kidney.

[52] Granger DN, Kvietys PR, Perry MA, Barrowman JA, Johnson LR (1987). The microcirculation and intestinal transport. Physiology of the Gastrointestinal Tract.

[53] Lipowsky HH, Kovalcheck S, Zweifach BW (1978). The distribution of blood rheological parameters in the microvasculature of cat mensentery. Circ Res.

[54] Delaney JP (1969). Arteriovenous anastomotic blood flow in the mesenteric organs. Am J Physiol.

[55] Kazmers A, Wright CD, Whitehouse WM, Zelenock GB, Lindenauer SM, Stanley JC (1981). Glucagon and canine mesenteric hemodynamics: effects on superior mesenteric arteriovenous and nutrient capillary blood flow. J Surgical Res.

[56] Sherman JL (1963). Normal arteriovenous anastomoses. Medicine.

[57] Saxena PR, Verdouw PD (1985). Tissue blood flow and localization of arteriovenous anastomoses in pigs with microspheres of four different sizes. Pflugers Arch.

[58] Krogstad AL, Elam M, Karlsson T, Wallin BG (1995). Arteriovenous anastomoses and the thermoregulatory shift between cutaneous vasoconstrictor and vasodilator reflexes. J Auton Nerv Syst.

[59] Kitajima M, Otsuka S, Shimizu A, Nakajima M, Kiuchi T, Ikeda Y, Oshima A (1988). Impairment of gastric microcirculation in stress. J Clin Gastroenterol.

[60] Rhodin JA (1967). The ultrastructure of mammalian arterioles and precapillary sphincters. J Ultrastruct Res.

[61] Sherf L, Ben-Shaul Y, Lieberman Y, Neufeld HN (1977). The human coronary microcirculation: an electron microscopic study. Am J Cardiol.

[62] Anderson BG, Anderson WD (1978). Scanning electron microscopy of microcorrosion casts; intracranial and abdominal microvasculature in domestic animals. Am J Anat.

[63] Anderson BG, Anderson WD (1980). Microvasculature of the canine heart demonstrated by scanning electron microscopy. Am J Anat.

[64] Nakai K, Imai H, Kamei I, Itakura T, Komari N, Kimura H, Nagai T, Maeda T (1981). Microangioarchitecture of rat parietal cortex with special reference to vascular “sphincters”. Scanning electron microscopic and dark field microscopic study. Stroke.

[65] Castenholz A (1983) Visualization of periendothelial cells in arterioles and capillaries by scanning electron microscopy of ultrasound treated and plastoid injected brains in rats. Scan Electron Microsc 1983:161–170 6356330

[66] He YY, Yu SJ, Cui Y, Du P (2010). Morphological study on microvasculature of left ventricular wall in infant and adult yaks. Anat Rec.

[67] Tsunenari I (1993). Cushion-like structure in coronary arteries of rats. Kaibogaku Zasshi.

[68] Higuchi K, Hashizume H, Aizawa Y, Ushiki T (2000). Scanning electron microscopic studies of the vascular smooth muscle cells and pericytes in the rat heart. Arch Histol Cytol.

[69] Shiraishi T, Sakaki S, Uehara Y (1990). Architecture of the medial smooth muscle of the arterial vessels in the normal human brain: a scanning electron-microscopic study. Scanning Microsc.

[70] Ushiwata I, Ushiki T (1990). Cytoarchitecture of the smooth muscles and pericytes of rat cerebral blood vessels. A scanning electron microscopic study. J Neurosurg.

[71] Sasaki S, Kobayashi N, Dambara T, Kira S, Sakai T (1995). Structural organization of pulmonary arteries in the rat lung. Anat Embryol.

[72] Sakai T, Kriz W (1987). The structural relationship between mesangial cells and basement membrane of the renal glomerulus. Anat Embryol.

[73] Elger M, Sakai T, Kriz W (1998). The vascular pole of the renal glomerulus of rat. Adv Anat Embryol Cell Biol.

[74] Hosoyamada Y, Sakai T (2012). Structural arrangement of collagen fibrils in the periarterial connective tissue of the kidney: their functional relevance as a structural stabilizer against arterial pressure. Anat Sci Int.

[75] Hosoyamada Y, Sakai T (2005). Structural and mechanical architecture of the intestinal villi and crypts in the rat intestine: integrative reevaluation from ultrastructural analysis. Anat Embryol.

[76] Hosoyamada Y, Sakai T (2007). Mechanical components of rat intestinal villi as revealed by ultrastructural analysis with special reference to the axial smooth muscle cells in the villi. Arch Histol Cytol.

[77] Fuxe K, Sedvall G (1965). The distribution of adrenergic nerve fibres to the blood vessels in skeletal muscle. Acta Physiol Scand.

[78] Schenk E, El Badawi A (1968). Dual innervation of arteries and arterioles—a histochemical study. Z Zellforsch.

[79] Lundvall J, Hillman J, Gustafsson D (1982). beta-Adrenergic dilator effects in consecutive vascular sections of skeletal muscle. Am J Physiol.

[80] Saltzman D, DeLano FA, Schmid-Schönbein GW (1992). The microvasculature in skeletal muscle: VI. Adrenergic innervation of arterioles in normotensive and spontaneously hypertensive rats. Microvasc Res.

[81] Norberg KA, Olson L (1965). Adrenergic innervation of the salivary glands in the rat. Z Zellforsch.

[82] Dahlström A, Fuxe K (1965). The adrenergic innervation of the nasal mucosa of certain mammals. Acta Otolaryngol.

[83] Oda M, Nakamura M, Honda K, Komatsu H, Kaneko K, Azuma T, Nishizaki Y, Tsuchiya M (1988). Involvement of autonomic nervous system in gastric mucosal defense mechanism. J Clin Gastroenterol.

[84] Mann R, Bell C (1993). Distribution and origin of aminergic neurones in dog small intestine. J Auton Nerv Syst.

[85] Itakura T, Yamamoto K, Tohyama M, Shimizu N (1977). Central dual innervation of arterioles and capillaries in the brain. Stroke.

[86] Higgins JC, Eady RA (1981). Human dermal microvasculature: I. Its segmental differentiation. Light and electron microscopic study. Br J Dermatol.

[87] Stingl J (1976). Fine structure of precapillary arterioles of skeletal muscle in the rat. Acta Anat.

[88] Holley JA, Fahim MA (1983). Scanning electron microscopy of mouse muscle microvasculature. Anat Rec.

[89] Risco JM, Nopanitaya W (1980). Ocular microcirculation: scanning electron microscopic study. Invest Ophthalmol Vis Sci.

[90] Morrison JC, DeFrank MP, Van Buskirk EM (1987). Regional microvascular anatomy of the rabbit ciliary body. Invest Ophthalmol Vis Sci..

[91] Yoneya S, Tso MO (1987). Angioarchitecture of the human choroid. Arch Ophthalmol.

[92] Pannarale L, Onori P, Ripani M, Gaudio E (1991). Retinal microcirculation as revealed by SEM corrosion casts in the rat. Eur J Ophthalmol.

[93] Pannarale L, Onori P, Ripani M, Gaudio E (1996). Precapillary patterns and perivascular cells in the retinal microvasculature. A scanning electron microscope study. J Anat.

[94] Bhutto IA, Amemiya T (1995). Retinal vascular changes during aging in Wistar Kyoto rats. Application of corrosion cast and scanning electron microscopy. Ophthalmic Res.

[95] Bhutto IA, Amemiya T (1995). Corrosion cast demonstration of retinal vasculature of normal Wistar-Kyoto rats. Acta Anat.

[96] Bhutto IA, Amemiya T (1997). Vascular changes in retinas of spontaneously hypertensive rats demonstrated by corrosion casts. Ophthalmic Res.

[97] Murakami M, Sugita A, Shimada T, Nakamura K (1979). Surface view of pericytes on the retinal capillary in rabbits revealed by scanning electron microscopy. Arch Histol Jpn.

[98] Nagai T, Morimitsu T, Nagai M, Tono T (1983). Surface view of strial vessel, prominence vessel, and external sulcus cells as revealed by scanning electron microscopy. Arch Otorhinolaryngol.

[99] Zhang JQ, Iijima T, Tanaka T (1993). Scanning electron microscopic observation of the vascular wall cells in human dental pulp. J Endod.

[100] Iijima T, Zhang JQ (2002). Three-dimensional wall structure and the innervation of dental pulp blood vessels. Microsc Res Tech.

[101] Lane BP, Zeidler M, Weinhold C, Drummond E (1983). Organization and structure of branches in the rat pulmonary arterial bed. Anat Rec.

[102] Fujiwara T, Uehara Y (1984). The cytoarchitecture of the wall and the innervation pattern of the microvessels in the rat mammary gland: a scanning electron microscopic observation. Am J Anat.

[103] Miller BG, Woods RI, Bohlen HG, Evan AP (1982). A new morphological procedure for viewing microvessels: a scanning electron microscopic study of the vasculature of small intestine. Anat Rec.

[104] Ohtani O, Lee MH, Wang QX, Uchino S (1997). Organization of the blood and lymphatic microvasculature of the gallbladder in the guinea pig: a scanning electron microscopic study. Microsc Res Tech.

[105] Pais D, Esperança-Pina JA (2001). Microvasculature of the corpus epididymis of canis familiaris. A scanning electron microscopic study of microvascular corrosion casts. Ital J Anat Embryol.

[106] Polguj M, Jȩdrzejewski KS, Topol  M (2011). Angioarchitecture of the bovine spermatic cord. J Morphol.

[107] Berman HJ, McNary W, Ausprunk D, Lee E, Weaver S, Sapawi R (1972). Innervation and fine structure of the precapillary sphincter in the frog retrolingual membrane. Microvasc Res.

